# Principal Criteria for Evaluating the Quality, Safety and Efficacy of hMSC-Based Products in Clinical Practice: Current Approaches and Challenges

**DOI:** 10.3390/pharmaceutics11110552

**Published:** 2019-10-24

**Authors:** Juan Antonio Guadix, Javier López-Beas, Beatriz Clares, José Luis Soriano-Ruiz, José Luis Zugaza, Patricia Gálvez-Martín

**Affiliations:** 1 Department of Animal Biology, Faculty of Sciences, University of Málaga, Instituto Malagueño de Biomedicina (IBIMA), Campus de Teatinos s/n, Málaga E-29071, Spain; jaguadix@uma.es; 2 BIONAND, Centro Andaluz de Nanomedicina y Biotecnología (Junta de Andalucía, Universidad de Málaga), c/ Severo Ochoa nº25, Campanillas, Málaga E-29590, Spain; 3 Andalusian Center for Molecular Biology and Regenerative Medicine (CABIMER), University of Pablo de Olavide-University of Seville-CSIC, Seville 41092, Spain; javicucha@gmail.com; 4 Department of Pharmacy and Pharmaceutical Technology, Faculty of Pharmacy, University of Granada, Granada E-18071, Spain; beatrizclares@ugr.es (B.C.); jlsoriano@correo.ugr.es (J.L.S.-R.); 5 Department of Genetics, Physical Anthropology and Animal Physiology, University of the Basque Country, Leioa E-48940, Spain; joseluis.zugaza@ehu.es; 6 Achucarro Basque Center for Neuroscience, Bizkaia Science and Technology Park, building 205, Zamudio E-48170, Spain; 7 IKERBASQUE, Basque Foundation for Science, María Díaz de Haro 3, Bilbao E-48013, Spain; 8 R&D Human Health, Bioibérica S.A.U., Barcelona E-08029, Spain

**Keywords:** mesenchymal stem cells, good manufacturing practice, cell characterization, release criteria and manufacturing process

## Abstract

Human Mesenchymal Stem Cells (hMSCs) play an important role as new therapeutic alternatives in advanced therapies and regenerative medicine thanks to their regenerative and immunomodulatory properties, and ability to migrate to the exact area of injury. These properties have made hMSCs one of the more promising cellular active substances at present, particularly in terms of the development of new and innovative hMSC-based products. Currently, numerous clinical trials are being conducted to evaluate the therapeutic activity of hMSC-based products on specific targets. Given the rapidly growing number of hMSC clinical trials in recent years and the complexity of these products due to their cellular component characteristics and medicinal product status, there is a greater need to define more stringent, specific, and harmonized requirements to characterize the quality of the hMSCs and enhance the analysis of their safety and efficacy in final products to be administered to patients. These requirements should be implemented throughout the manufacturing process to guarantee the function and integrity of hMSCs and to ensure that the hMSC-based final product consistently meets its specifications across batches. This paper describes the principal phases involved in the design of the manufacturing process and updates the specific technical requirements needed to address the appropriate clinical use of hMSC-based products. The challenges and limitations to evaluating the safety, efficacy, and quality of hMSCs have been also reviewed and discussed.

## 1. Introduction

Several aspects of human Mesenchymal Stem Cells (hMSCs) have been widely studied in recent decades, including their origin, methods for obtaining them, their differentiation, safety, efficacy, mechanism of action, etc. [1]. Due to their multipotent potential, ease of harvesting and in vitro expansion, differentiation into multiple cell lineages, as well as their regenerative and immunomodulatory properties and ability to migrate to the exact area of injury [2], hMSCs are of great interest for the development of new hMSC-based products in advanced therapies and regenerative medicine. They have a wide range of therapeutic applications and are quickly becoming a valuable tool for different pathologies such as cardiac, neurological and autoimmune diseases, skin lesions, cancer, etc. [3,4,5]. 

Currently, more than 500 clinical trials on hMSC-based products are being conducted to evaluate their safety and side effects, define their doses and routes of administration, and to determine their efficacy as therapeutic agents in specific targets. However, only eight hMSC-based products have been granted marketing authorization [6]. This is primarily due to the complexity of these products in terms of the characteristics of their cellular components and their legal consideration as pharmaceutical products [7]. Regarding the cellular components, hMSCs must be living when they are administered to maintain their functionality and integrity. As for their legal status, the leading health agencies classify them as medicinal products or biologic products. This status means that, before hMSCs can be administered, an hMSC-based product must be approved by the competent authority, either as an investigational medicinal product to be administered during a clinical trial or granted marketing authorization [8]. In both cases, the results of all preclinical and clinical studies, respectively, must be collected, a description of the manufacturing process must be prepared, and they must both be submitted to the regulatory authorities [9,10].

Therefore, properly designing a manufacturing process is critical to being able to authorize an hMSC-based product. In the design of a manufacturing process, specific technical requirements must be implemented, through which the quality, safety and efficacy of the final product to be manufactured can be guaranteed before it is administered in humans as a therapeutic agent. The manufacturing process design must be validated, guaranteeing that all the hMSC-based products, manufactured using the same protocol, meet the same specifications from batch to batch. It should be noted that for autologous hMSC-based products, each process yields a single product, that is to say, one batch. However, for allogeneic hMSC-based products, each process can yield multiple, but still finite, batches.

According to the latest advances in basic and preclinical research, new analytical techniques and methods to evaluate hMSC-based products have been proposed [11,12]. Furthermore, in recent years, regulatory agencies like the Food and Drug Administration (FDA) and European Medicines Agency (EMA) have published specific guidelines to define the criteria for cell-based products, which must be implemented in the manufacturing of hMSC-based products. This highlights the need to define and update the specific technical criteria to design an hMSC-based product manufacturing process in compliance with the current legal requirements [13,14,15].

Therefore, this review describes the most critical stages involved in the design of the hMSC-based product manufacturing process and updates the minimum requirements for guaranteeing the clinical use of the hMSC-based products. Furthermore, the current challenges and limitations to evaluating the quality, safety and efficacy of hMSCs for administration in humans as therapeutic agents have also been reviewed and discussed.

## 2. Design of an hMSC-Based Product Manufacturing Process 

An hMSC-based product manufacturing process should be carried out under the same conditions as for a conventional medicine [9,16], that is, under the conditions set out by the current Good Manufacturing Practice (GMP) guidelines [17]. GMP ensures that hMSC-based products are consistently manufactured and checked according to quality standards, which must be previously defined and approved in a quality control program. On the other hand, GMP minimizes the risks of contamination by infectious agents during the cellular manufacturing process. This requires the predetermination of a risk assessment protocol for each process. Together with the quality control program and risk assessment protocol, other manufacturing protocols should be in place and validated together with the facilities, such as in vitro expansion process, cleaning, environmental monitoring, transportation, disinfection of materials, media-fill, etc. The staff must also be qualified for their assigned activities [18,19]. For an optimal implementation of all these protocols, designing a system based on standard operating procedures (SOPs) is highly recommended. Once the hMSC-based product manufacturing process is defined, manufacturing authorization is required for each process by the competent authorities [13,15].

**Figure 1:**
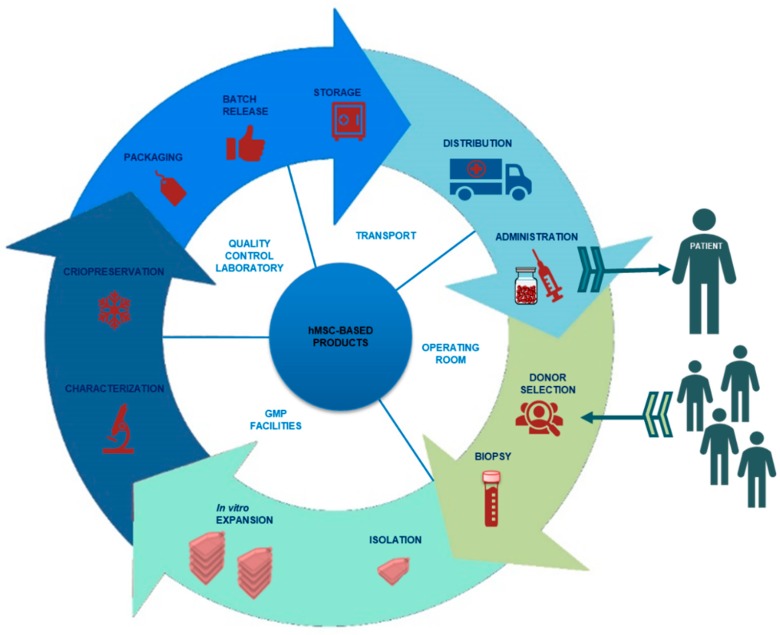
Diagram of all phases involved in the manufacturing of a human Mesenchymal Stem Cell (hMSC)-based product for clinical administration and where they are performed.

The hMSC-based product manufacturing process encompasses different stages: selection and evaluation of donor suitability (allogeneic therapies), serologic testing, biopsy, isolation, processing of the biological sample from which the defined cellular dosage will be obtained through an in vitro expansion process if necessary, characterization of the intermediate products, and final product, packaging and release (Figure 1). 

### 2.1. Donor Selection and Serology

The first step in the design of an hMSC-based product manufacturing process is the selection of the donor (allogeneic products), and then checking the biological starting material through donor testing for both autologous and allogeneic hMSC-based products. Each donor must undergo a physical examination to rule out signs or symptoms of communicable diseases. In addition, a serological study should be performed to detect at least the human immunodeficiency virus (type 1 and type 2), hepatitis virus (B and C) and Treponema pallidum [20]. The FDA also requires some relevant diseases such as West Nile Virus, sepsis and vaccinia to be ruled out [21,22]. 

### 2.2. Isolation and Processing of hMSCs

In many cases, the clinical application of hMSC-based products involves the administration of a large number of cells, depending on the dose to administer. It is well known that hMSCs are easily isolated from a variety of human tissues, including bone marrow, umbilical cord blood and adipose tissue [23,24]. However, hMSCs isolation techniques can vary according to the cell origin. Therefore, is necessary to develop hMSCs isolation and in vitro expansion protocols to obtain appropriate cell doses. Several techniques have been described for hMSCs isolation, including direct plating, red blood cell lysis and gradient density [25]. Bone marrow derived stem cells are often designated as the gold standard. However, given the low percentage of hMSCs obtained from bone marrow aspiration or umbilical cord blood, it is remarkable that the density gradient centrifugation method of adipose tissue constitutes a simple and cost effective way to increase the MSCs yield by a minimally invasive procedure [26,27].

Consequently, hMSCs must undergo in vitro expansion by means of long-term cultures, allowing a large-scale production. The in vitro expansion process involves substantial manipulation of hMSCs, which entails an important modification of the biological characteristics, physiological functions and structural properties of cells [28]. This stage is considered critical, since any minimum alteration could affect the intrinsic characteristics of cells [29]. Therefore, it will be crucial to control the entire process. In addition, reagents involved in this phase must be controlled. In vitro expansion of hMSCs using growth supplement such as fetal bovine serum (FBS) involves potential risk such as zoonotic infectious agents, microbial contaminants, and immunological reactions. Human platelet lysate (hPL) can be used as a suitable alternative to FBS in clinical grade manufacture of cell therapies. The hPL for hMSCs proliferation under animal serum-free conditions could be obtained from whole blood-derived pooled platelet concentrates or the platelet apheresis method after positive red blood cell antibody screening [30]. The risk of transmission of human blood pathogens as viruses, bacteria, fungi and prions, obliges to perform a virus-inactivation treatment during hPL production to ensure optimal safety. In this regard, several methods have been proposed for pathogen inactivation, such as freeze–thaw cycles (−80/+37 °C), heat-treatment (56 °C), serum-conversion by calcium chloride, solvent/detergent (S/D) treatment or psoralen/UV treated human serum [31,32,33].

### 2.3. Characterization of hMSCs 

The cellular component present in each hMSC-based product should be defined, monitored, and controlled through standardized and consistent methods based on accumulating data, to guarantee the quality, safety and efficacy of the final product [34,35]. 

**Figure 2:**
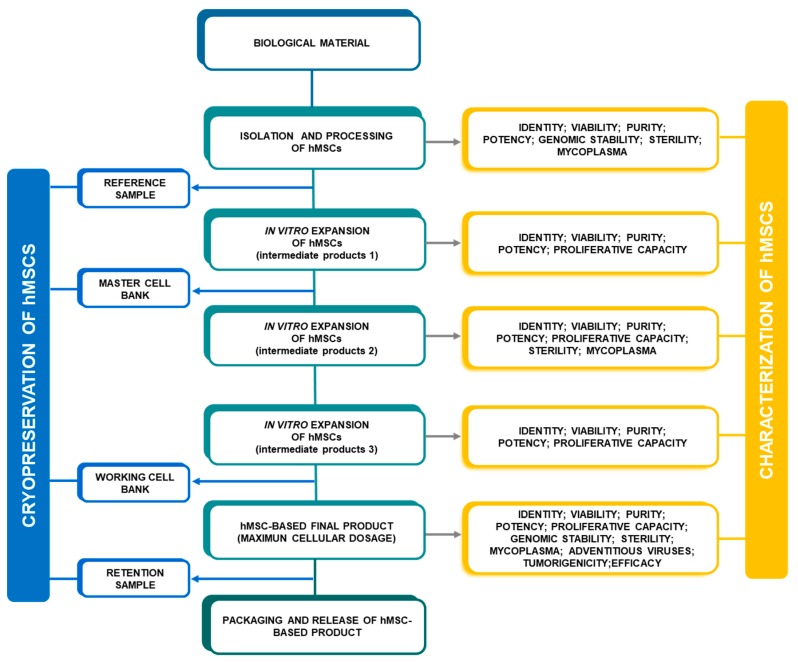
Example of an outline of the quality control program for the development of clinical-grade mesenchymal stem cells during the validation phase.

The characterization of hMSCs should be carried out based on their most critical attributes according to the legal requirements previously established by health authorities. The evaluation of identity, viability, purity, potency, proliferative capacity, genomic stability, tumorigenicity and efficacy are considered minimum criteria for the characterization of hMSCs [14,17,36,37]. Besides, the specific GMP guidelines indicate that cells should be monitored during the in vitro expansion process to analyze for any contamination of the product [38,39]. Hence, microbiological quality testing in terms of sterility, mycoplasma and viruses should be carried out to ensure that hMSC-based products are not infected after manufacturing. Throughout the in vitro expansion process, hMSCs can be characterized at different points such as the isolation stage, reference sample, intermediate product, final product, retention sample, master cell bank (MCB), and working cell bank (WCB; when necessary according to the process) (Figure 2). 

### 2.4. Cryopreservation of hMSCs

hMSCs can be administered fresh or frozen for later use, such as in cases of allogenic therapies. Currently, more than one third of MSC-based clinical trials use cryopreserved cells [40]. In addition, reference and retention samples, MCB and WCB are cryopreserved before being stored. Thus, aspects such as cryopreservation and defrosting methods, maximum cryopreservation time and the process to activate cells are crucial and should be validated and defined in the manufacturing process. 

Cryopreservation techniques are based on the ability of small molecules to enter cells and prevent formation on intracellular ice crystals and osmotic effects to preserve cells for future use, generically called cryoprotective agents (CPAs). The most commonly used freezing medium for hMSCs is based on dimethyl sulfoxide (DMSO) as CPA to lower their freezing point [41],and animal serum or animal protein-free cryopreservation media (CryoStor), to preserve the functionality and viability of MSCs during a slow freezing process (between 1 and 2.5 °C/min) [42,43]. In order to maximize cell survival, an optimal cooling rate is normally achieved by using a controlled-rate freezer (CRF) with a cryocell system. The cryoprotective effect preserves most in vitro cell characteristics and functional potency in a short acclimation period post-thaw and they should not take more than a few days to grow robustly [44,45,46,47]. Rather, the cell density of cryopreserved hMSCs depends on downstream application. Most of clinical protocols recommend the intravenously administration of one to two million cells per kilogram of patient’s weight or up to 120–600 million cells injected subcutaneously or endomyocardially [8,48,49]. Thus, hMSCs are usually frozen in cryovials in a total volume of 1 mL per cryovial at a concentration of 2–25 million cells/mL. 

### 2.5. hMSC-Based Product Packaging

Once the manufacturing process has been defined, the packaging of the hMSC-based final product must be determined, defining i) the primary packaging (e.g., bag or syringe) according to the manufactured dose forms and administration route and ii) secondary packaging (e.g., label, box, leaflet). 

Also, during this phase, stability testing should be implemented in order to evaluate and define the optimal storage conditions for the final product, in terms of temperature, throughout its storage and distribution, to keep its specifications unchanged [50]. The stability test also makes it possible to calculate the shelf-life of the hMSCs in the final product, which is formulated from hMSCs, excipients, other types of cells, and even in combination with medical devices. The shelf-life is defined as the maximum period of time that an hMSC-based final product can be stored, maintaining its properties prior to administration to the patient [51]. This test should be performed according to the standards described by International Conference on Harmonization (ICH) guidelines, Quality of Biotechnological Products: Stability Testing of Biotechnological/Biological Products Q5C, ICH 1995, whenever possible [52].

### 2.6. Release of hMSC-Based Products

The last phase in the manufacturing process of an hMSC-based product is its release according to requirements established in the product specifications. The specification sheet precisely describes the acceptance criteria that an hMSC-based product must meet, based on previous studies. It also defines the analytical techniques and methods for analysis [53].

The evaluation of identity, viability, purity, potency, proliferative capacity, genomic stability, and microbiological testing are mandatory quality controls for hMSC-based final products and should be established in a specification sheet (Table 1). In the case of the sterility test in hMSC-based products, the final results might not be available before their administration, since it is not always possible to obtain a frozen product, and fresh hMSC-based final products must be administered within 48–72 h [54]. Thus, the in-process testing performed on intermediate products will be critical to guarantee optimal product release (first release). Once all the results have been obtained, the final release is made, which is an administrative release since the hMSC-based product has already been administered.

Before the final product release, a substantial aliquot should be cryopreserved (retention sample) as a back-up for reanalysis. Then, hMSC-based products can be stored and/or sent for administration. These last phases must also be controlled, ensuring good distribution practices (GDP) [55].

## 3. Minimal Criteria for hMSC Characterization

**Figure 3:**
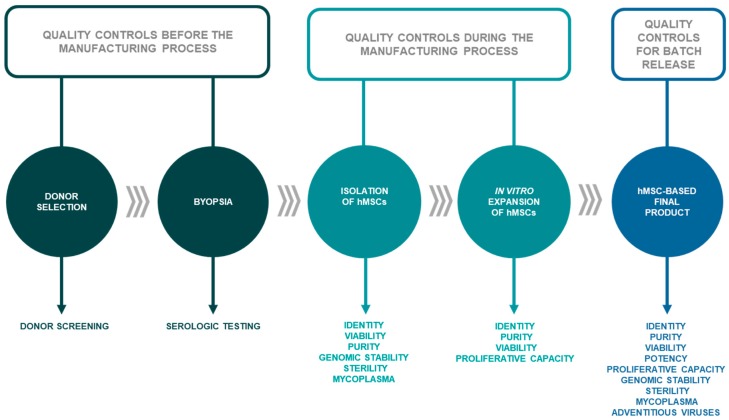
Quality controls to be carried out before the in vitro expansion process, in the intermediate product and in the final product.

Throughout the manufacturing process, different quality controls must be carried out, evaluating both the biological samples, the hMSC-based intermediate products, and the hMSC-based final product before being released (Figure 3). 

### 3.1. Identity

The objective of identity assays in hMSC-based products is to guarantee that the cellular component is really hMSC-based by verifying that there is no cross-contamination with another cell type. With the identity assay, it is possible to distinguish between different cell types used during the manufacturing process or other cell products that can be manufactured in the same GMP-certified facilities.

To help identify hMSCs, the International Society of Cellular Therapy (ISCT) proposed three minimum criteria in 2006: i) MSCs must be plastic-adherent (appearing under the microscope as fibroblast cells); ii) MSCs must express CD73, CD90, CD105, Oct-4, Rex-1, Sox-2, and there must be an absence of expression of CD45, CD34, CD14 or CD11b, CD79 alpha or CD19, and human leukocyte antigen (HLA)-DR surface molecules; iii) MSCs must have a high plasticity to differentiate to adipocytes, chondroblasts, and osteoblasts under standard in vitro culture conditions [56,57]. These characteristics can be evaluated by microscopy, immunophenotypic characterization and cell differentiation tests, respectively. Minimal criteria proposed by ISCT consider HLA-DR expression as a negative marker. However, its expression is largely unpredictable during clinical-grade large-scale hMSC in vitro expansion. Therefore, HLA-DR expression should be considered as informative about the quality of hMSCs for clinical use rather than as a criterion to hMSCs identity [58,59]. 

The cell differentiation capacity of hMSCs is evaluated by specific staining. Von Kossa or Alizarin Red staining are used to examine the osteogenic differentiation through calcium deposition, Oil Red O staining evaluates the adipocyte differentiation through the presence of lipid droplets and Alcian Blue staining is used to show the chondrogenic differentiation through cellular aggregates floating freely in suspension in the culture [60]. When hMSCs are cultured in shared spaces or processed with the same instruments for different donors, it is advisable to perform a Short Tandem Repeat (SRT) DNA profiling analysis [60].

### 3.2. Viability

A viability assay can determine the number of living hMSCs present in a culture or product. The study of viability in hMSC-based products establishes the percentage of viable hMSCs, which must be kept alive at time of administration, since the functionality of these products depends on living cells, even though in certain cases dying/senescent cells must be considered since these cells can secrete relevant cytokines with high anti-inflammatory potential. Furthemore, this assay is performed throughout the entire in vitro expansion process, analyzing the intermediate products generated in each passage.

The cell-count procedure may be performed manually by the dye exclusion test, separating the viable from the non-viable cells, because only dead or damaged cells absorb the dye and are stained (hemocytometer or Neubauer chamber). The most commonly used stains are trypan blue, propidium iodide, calcein-AM, erythrosin B and nigrosin. Furthermore, different automated methods can be used (particle counter or flow cytometer) [61]. Automated methods can also be used, such as flow cytometry or by specific equipment. However, they must be previously validated.

Cell viability (*V*) is expressed as a percentage and is calculated using the expression *V* = *n*/*N* × 100, where *n* is the number of total viable cells (unstained cells) and *N* is the number of total cells (stained and unstained cells).

The minimum acceptance criteria for the viability assay in hMSC-based products are viability ≥80% during different stages of cell culture, intermediate product and final product, and viability ≥70% after cell thawing process from liquid N_2_ [62,63].

### 3.3. Purity

The purity study guarantees that hMSC-based products are free of any undesirable impurity or foreign material, whether it is harmful to patients or not. Different types of impurities can be found, which can originate from product-related or manufacturing process-related impurities.

#### 3.3.1. Cell Impurities

After biopsy and cell isolation, it is necessary to rule out the presence of other plastic-adherent cell populations like hMSCs. The primary types of plastic-adherent cells are monocytes, macrophages, fibroblasts, etc. Their immunophenotypic characterization helps us determine the percentage of different cells that have been collected. Monocytes and macrophages are characterized through CD14, CD34, and CD45 gene expression, and although they have a fusiform appearance, these cells have a larger diameter (30–40 µm). Fibroblasts also express CD34 in their inactivated state. Acceptance criteria to work with hMSC cultures require cells expressing CD14, CD20, CD34, and CD45 not exceeding 10% of the total analyzed cell suspension [56,64]. The purpose of the above is to minimize the impact of these cell strains on the efficacy/safety of hMSC-based final products.

#### 3.3.2. Process Impurities

Different reagents are used throughout the in vitro expansion process for hMSCs. Due to the safety risks of these reagents, they must not be present in the final product, as antibiotics and reagents of animal origin. Fetal bovine serum (FBS) and trypsin are the primary reagents of animal origin used in the manufacturing phase of hMSC-based products. The EMA published specific guidelines for the use of the FBS and the porcine trypsin [65,66]. FBS is a sterile serum, suitable for cell culture for use in humans. It was recommended by the EMA in the list of FBS with certificate Transmissible Spongiform Encephalopathy risk, Certificate of Suitability from the European Directorate for the Quality of Medicines. This serum is characterized by the absence of pathogenic viruses because it originates from a bovine spongiform encephalopathy-free zone (Australia).

Other undesirable toxic effects are related to the use of DMSO, which is used for cellular cryopreservation at a maximum final concentration of 10%, because higher concentrations could produce cell damage. After thawing, DMSO activity is inhibited by a 1/10 dilution in 10% FBS medium. Subsequently, cells are centrifuged and the supernatant containing DMSO is aspirated and removed when the process allows it. hMSCs are grown in vitro and subjected to several passes and culture medium changes, whereby at the end, the concentration of DMSO is well tolerated, with no observable toxic effects at concentrations of 0.1% (*v*/*v*) or lower [67].

Other residual contaminants that should be detected after the in vitro expansion process for hMSCs are antibiotics, due to their frequent use. Although hMSC cultures are carried out in sterile conditions (GMP grade), it is necessary to minimize the possibilities of contamination by adventitious agents.

Contamination by extrinsic material can cause toxic reactions; therefore, it is essential to determine the absence of any pyrogens in hMSC-based products before being administered to patients. The main assays for studying the purity of these products are the pyrogenicity and endotoxin tests. Endotoxins are lipopolysaccharides from Gram-negative bacteria, and their presence in any product will imply contamination by pyrogenic components. The main method for the detection of endotoxins is the Limulus Amoebocyte Lysate (LAL) test. However, automated methods can also be used [68].

### 3.4. Potency

Potency is defined by the FDA as “the specific ability or capacity of the product, as indicated by appropriate laboratory tests or by adequately controlled clinical data obtained through the administration of the product in the manner intended, to effect a given result” [69]. The pursuit of suitable and reliable potency assays is an important requirement to obtain consistent and safe cellular products, and ultimately products capable of exercising effective therapeutic effects.

Further acknowledgement to understand different mechanisms of action (MoA) of hMSCs has become one of the main challenges for advancing hMSC-based therapies [70]. The complexity normally associated with hMSC-based products can be a substantial challenge in the implementation of potency assays. Most of the effects and MoA of hMSCs are paracrine and/or trophic effects [71]. Furthermore, hMSC populations share essential MoA related to the tissue source and/or in vitro expansion that mediate the immunomodulatory function and therapeutic potential. Therefore, the manufacture of hMSC-based products focuses on the most well-known biological properties of hMSCs—pro-angiogenic and immunomodulatory effects [72]. The establishment of adequate potency assays is imperative for researchers and regulatory agencies to predict the therapeutic efficacy of hMSC-based investigational medicinal products or products with marketing authorization.

To address this issue, the ISCT considers immune functional assays for hMSCs as a potency release requirement for hMSC-based products [73]. The conventional approach emphasizes the development of quantitative biological methods to determine the potency of cellular products related to their specific capacity to exert a given effect and meet the requirements imposed by regulatory authorities. However, it is unlikely that one single assay will capture all biological effects. Therefore, various biological assays may be needed to completely define potency. 

The criteria demanded by ISCT to define hMSCs seem to be insufficient predictors to guarantee therapeutic success. In this regard, a potency assay is an indispensable tool to ensure that hMSC-based products exert a differential effect at a specific dosage. In an effort to improve the methods to ascertain potency of hMSCs for clinical use, some traditional potency assays have been proposed, which have included anti-inflammatory and immunomodulatory potency assays such as endothelial tube formation (MultiStem^®^) [74]; Ovalbumin challenge [75]; angiogenic potency assays (i.e., secretion of “pro-angiogenic factors” such as VEGF, IL-6, PDGF, CXCL5, etc.) [74,76,77], IL-10 release [78], measure of secretion TNF-α stimulated gene/protein 6 (TSG-6) [79]; and anti-inflammatory soluble mediators such as indoleamine 2,3-dioxygenase (IDO), prostaglandin E2 (PGE2), transforming growth factor beta (TGF-β), nitric oxide, HLA-G5, and several interleukins (ILs), which act via a paracrine effect [80,81,82,83,84]. More recently, innovative assays have been developed and validated based on the measurement of functionalities related to in vitro expansion (i.e., gene expression analysis, telomere length, cell size and self-renewal properties) [85], protein-based assay of secretome, and the study of significant surface markers by flow cytometry [86]. 

In order to confirm that results are reliable and robust, it is critical to include an adequate control test with appropriate reference-standard materials. Regarding the potency assay selection, an adequate development should include appropriate control tests including optimal references such as a “cell ruler” to compare final batches [87]. 

### 3.5. Proliferative Capacity

Proliferation is an essential feature of stem cells for self-renewal and expansion, as well as for defining their degree of stemness [88]. Considering that the number of hMSCs in fresh tissue (e.g., bone marrow or adipose tissue) is not sufficient to have therapeutic potency, clinical applications of hMSCs depend fundamentally on their ability to replicate in long-term in vitro expansion. An extensive in vitro expansion following isolation is always required to generate a sufficient amount of cells for hMSC-based products [89]. The Cell Therapy Working Group recommends Population Doubling (PD) as an accurate method to estimate cell growth and to define the time of cells in culture [90,91]. The in vitro expansion growth rate of hMSCs should be calculated through cell counting for each passage. The result should be shown as Cumulative Population Doublings (CPD) according the equation log *N*/log 2, where *N* is the final number of confluent cells divided by the initial number of cells seeded [92]. The CPD determine the number of divisions that a cell has undergone during a process. The CPD will depend on various factors such as the species, age of donor, origin of biopsy, culture conditions and time of in vitro expansion. The process of hMSC culture expansion can vary greatly depending on the cellular age. PD establishes an accurate method, which ensures the use of young cell cultures with high proliferation and multilineage capacity. Proliferative capacity of cryopreserved cells can be maintained after several years [93] and hMSCs can be expanded with a significant clinical outcome in a few weeks. However, successive passages of hMSCs during long-term in vitro expansion (between 24 and 40 PD) might cause cells to undergo biological alterations in these expanded populations [94]. Prolonged expansion attenuates the proliferation rate and stemness maintenance of hMSCs. At later passages, a gradual proliferation delay and loss of multipotency contributes to reduced therapeutic potential [95,96]. Taking into account the gradual deterioration of hMSCs with long-term expansion, clinical recommendations for therapeutic use of hMSCs suggest their use in early passages (between three and seven) [97,98,99]. 

### 3.6. Genomic Stability 

Long-term in vitro culture of hMSCs entails risks that may be associated with the manufacturing process [89,100], due to immunogenicity and hazardous components of the medium, which can result in the presence of transformed cells with chromosomal aberrations [101,102,103,104]. Some authors have reported evidence that hMSCs are genetically stable for long-term culture [105], but there are scientific results demonstrating that in vitro culture of hMSCs may result in spontaneous transformations which can cause chromosomal instability [101,106,107,108]. These circumstances represent a potential risk for the clinical use of hMSCs; therefore, the genetic stability of these cells must be analyzed to ensure the safety of patients before their treatment [108,109]. 

In order to determine whether the expansion process is able to maintain the genetic stability of cells, and in parallel with the in vitro culture phase, the karyotype of the hMSC-based product should be studied [60].

The absence of chromosomal anomalies should be studied by karyotype analysis either by molecular methods, such as fluorescence in situ hybridization (FISH), spectral karyotyping (SKY), single nucleotide polymorphism (SNP) array, and microarray-based comparative genomic hybridization (aCGH), also called molecular karyotyping, or by conventional methods as Giemsa banding (G-banding), 4’-6-diamidino-2-phenylindole (DAPI) banding or QFQ-banding [108,110]. Some studies have shown that hMSCs maintained their genetic stability during the whole in vitro culture phase [111,112]. Tarte et al. [113] showed that the appearance of chromosomal alterations in expanded hMSCs was not related to the culture process, but that they could be due to inherent characteristics of the cells or to the biopsied sample. In any case, all of them can be associated with the senescence of the culture. The analysis by Ben-David et al. [114] revealed that after analyzing 132 samples of hMSCs, the probability of observing chromosomal alterations is 4%. In 2014, Capelli et al. [115] analyzed samples of clinical grade bone marrow-derived hMSCs by QFQ-banding. The results showed that spontaneous non-clonal anomalies were detected in 14 samples and clonal anomalies were only observed in three of the samples. These clonal anomalies were not associated with mutagenic problems [115]. These results are in line with the recommendations of the European Cell Products Working Party, which suggested, at a meeting held in October 2011, the presence of less than 10% of non-clonal anomalies and the absence of clonal anomalies in all studied metaphases, in comparison with the results of the karyotype in the retention sample, as release criteria for hMSC-based products, in order to determine the donor/patient status. According to general guidelines and quality assurance for cytogenetics, 20 metaphases should be examined, in cases where there are chromosomal alterations, an additional 20 metaphases should be studied [111,116,117]. The result should be indicated according to the International System for Human Cytogenetic Nomenclature [91,115].

### 3.7. Microbiological Quality Control

The safety of stem cell-based therapy underlies the demonstration that hMSC-derived products do not contain microorganisms like bacteria, fungi, mycoplasma, viruses, or even parasites and prions. Microbial contamination of hMSC-based products is one of the main causes of morbidity in recipients [118]. Therefore, facilities and personnel, as well as the reagents and raw materials involved in the hMSC-based product manufacturing process, must be validated and controlled. On the other hand, different tests, such as sterility testing, mycoplasma, and adventitious virus detection, should be carried out in the intermediate and final product to ensure that the hMSC-based products are not contaminated and to determine that the evaluated product is safe. 

#### 3.7.1. Sterility Test

An hMSC-based final product must be sterile, but due to size of hMSCs, a method like filtration cannot be carried out, nor is it possible to sterilize by heat or radiation because the viability of hMSCs would be affected. Therefore, the hMSC-based product manufacturing process must be performed under aseptic conditions. The aseptic processing must be validated using a process simulation test also known as media fill [19,119].

The recommended method for evaluating sterility of hMSC-based products is by direct inoculation of the sample into the growth media. This test requires an incubation step for 14 days [120]. This incubation period exceeds the short shelf-life (48–72 h) of the hMSC-based product (if it is not cryopreserved) [50]. Therefore, the administration will be done before sterility test results are obtained. New published guidelines for cell-based products recommend introducing alternative rapid tests to evaluate sterility before the release of the final product, for example, the BacT/ALERT test, automated growth-based methods or any other alternative methods [15,121,122,123]. Additionally, the intermediate products can be analyzed by stabilizing a control test before the release of the final product.

The selected sterility test methodology must be verified, ensuring that the analysis of the hMSC-based product does not interfere with the growth media used for the assay of microorganisms (growth promotion testing of culture media).

#### 3.7.2. Mycoplasma

Mycoplasma contamination of hMSC-based products can be caused by contaminated raw materials, reagents, or biologic samples (biopsy), or even be caused by the staff, facilities, and environment, since the manufacturing process is so complex. That said, these risks are supposedly prevented by aseptic manufacture. The traditional method for detecting mycoplasma requires a cultivation time of at least 28 days [124,125]. Alternatively, a semi-quantitative PCR reaction, rapid methods like Nucleic Acid Amplification Tests (NAAT), or indicator cell culture techniques should be carried out to determine if the hMSC-based final product is contaminated with mycoplasma before it can be released [122].

#### 3.7.3. Adventitious Viruses

The risks of contamination by adventitious viruses (enterovirus, adenovirus, human cytomegalovirus, and Epstein–Barr virus) represent an important safety concern that should be addressed in depth. Regulatory authorities, such as the EMA, FDA and the WHO have published specific guidelines reporting recommendations for adventitious agent testing [38]. Hence, this assay is essential for evaluating the safety of cells in every manufactured hMSC-based product by using hMSCs and unprocessed bulk fluid hMSCs. At the end of the manufacturing process, the product will be examined for viral cytopathogenic effects (CPE), hemagglutination (HA) or hemadsorption (HAD) [126,127,128]. 

The use of reagents of animal origin could introduce a risk of transmission of diseases or adventitious agents (e.g., prions), which implies a potential risk in the manufacturing process. For this reason, it will be necessary to certify that these reagents are free from any contaminant or adventitious viruses [39]. 

To perform the assessment of traditional adventitious viruses in hMSC-based products and to ascertain the lack of viruses before treatment, it will be necessary to have a sufficient quantity of product, which in many cases, is not easy to obtain. This involves the need for developing more advanced methods to detect adventitious viruses with improved efficiency, sensitivity and specificity of currently available methods such as retroviral reverse transcriptase, detection of viruses in cell culture and animal host systems or electron microscopy [127]. PCR-based methods and advanced nucleic acid-based technologies, namely high-throughput sequencing (HTS) or next-generation sequencing (NGS), afford a wide array of opportunities for patient safety and product efficacy [129,130,131]. These in vitro tests provide quantitative methods for evidencing infectious agents (adenovirus, enterovirus, cytomegalovirus, and Epstein–Barr virus) for the purpose of achieving a rapid release of hMSC-based products [129].

### 3.8. Tumorigenicity

One of the major concerns related to the use of hMSCs in regenerative medicine is the stem cells’ potential to become tumorigenic. Tumorigenicity is defined by the EMA as “the capacity of a cell population administered to an animal model to produce a tumor by proliferation at the site of administration and/or at a distant site by metastasis” [132]. The main difference between tumorigenicity and oncogenicity is that in tumorigenicity, the inoculated cells grow into tumors, and in oncogenicity, oncogenic agents transform cells of the injected species into neoplastic cells that grow into tumors. hMSCs are not commonly associated with tumorigenicity. To date, no evidence of malignant transformation has been reported in human patients after hMSC administration, and thus it can be considered a potential candidate for regenerative medicine clinical research [111]. However, there is a controversy regarding the transformation of hMSCs, due to the original observations of tumor formation in isolated hMSCs, which could be related to contamination, in particular through cross-contamination with malignant cells [133,134]. Hence, controversy exists over whether hMSCs themselves are tumorigenic. Recently, reports have emerged describing that long-term in vitro expansion of hMSCs can lead to molecular changes resulting in the acquisition of replicative senescence and promote tumorigenic risk [135]. During cellular senescence, hMSCs are able to secrete factors that increase the inflammatory response and promote either proliferation or migration of cancer cells such as proteases known as the senescence-associated secretory phenotype (SASP), growth factors, chemokines and cytokines [136,137]. 

Replicative senescence is a process that occurs from the beginning of the clinically expanded hMSCs and will progress with each passage during in vitro expansion. Tumorigenic potential of hMSCs may be related to the genomic instability of the cells, which may result in aberrant morphology or growth kinetics [138]. With that in mind, the evaluation of tumorigenicity of the finished products is essential to avoid the development of tumors, ectopic tissue formation, and/or malignant transformation. Despite the importance of carrying out such tests, no detailed guidelines about tumorigenicity testing for hMSC-based products in regenerative medicine and advanced therapies have been published. In this regard, we recommended monitoring cell growth as long as possible to detect any genetic instability leading to aberrant cell growth and to perform senescence assessments. According to current guidelines, the tumorigenesis process should be evaluated during the validation stage for a hMSC-based product to analyze chromosomal stability and cellular senescence.

### 3.9. Efficacy

The confirmation of efficacy and safety of cellular products is a major concern in cell therapies. The clinical use of hMSCs has a series of risks such as immune reactions, tumors, and/or growth of foreign tissue [139], which require close monitoring. One challenging aspect in early stages of clinical trials is to define accurate endpoints to show that hMSC-based products have safe and beneficial therapeutic effects in an increasing number of patients. 

Therapeutic efficacy of hMSC-based products has been observed in several applications [140]. After administration to patients, homing efficiency and selectivity are crucial factors for the success of cell-based therapies [141]. However, despite the fact that many hMSC-based therapies have shown clear benefits in ischemic and immune-related disorders [142,143], other clinical trials have failed to show a clear beneficial effect [144]. Therefore, it would be interesting to determine the clinical efficacy by adequate potency assays, prior to administration, and improve clinical outcomes of hMSC-based therapies during different phases of clinical trials, i.e., cell polarization for an adequate expression of pro-inflammatory mediators, critical for therapy for ischemic, inflammatory, or immune-related disorders [145]. In this same context, the evaluation of a Clinical Indications Prediction (CLIP) scale based on immunomodulatory, anti-inflammatory and angiogenic activity, trilineage differentiation, Colony-Forming Unit-Fibroblast (CFU-F) activity potential, survival and transcription factor TWIST1 levels, correlated with growth rate [146] could predict the therapeutic efficacy of different hMSCs.

## 4. Conclusions and Future Perspectives

Currently, hMSC-based products are considered potential therapeutic agents in specific targets as diabetes, burns, osteoarthritis, graft versus host disease, etc., so the clinical use of these products is arousing great interest in the biomedical field. Based on their status as a medicinal or biological product, these products must be manufactured and analyzed through a strict, robust, specific, and standardized process under GMP guidelines. In addition, specific quality control systems, highly trained staff and appropriate and monitored facilities are required. 

In recent years, the quality criteria that hMSC-based products must meet to be administered in humans have been intensified according to the greater knowledge of the safety profile and efficiency of hMSCs, the optimization of new analytical techniques, and the development of automated methods. Based on these advances, it is necessary to implement more restrictive and specific requirements throughout the hMSC-based product manufacturing process. All of them have triggered that the specification of final product will be update according the current criteria. In terms of quality, an hMSC-based product is considered safe and effective for release after confirming that the hMSCs are viable, are with fusiform morphology, do not form aggregates, are free of infectious agents, lack impurities, are at an optimal dosage, and are genetically stable.

On the other hand, the implementation of new and specific technical requirements entails the increase of cost of the manufacturing process, which has a direct impact on the price of hMSC-based products. Therefore, these products can reach very high prices in the healthcare system today. For this reason, it will be necessary to analyze their benefit-cost rates coupled with benefit-risk, determining their cost-effectiveness as potential treatment, and determining whether universal coverage of healthcare will be possible. Thus, cost-benefit ratio for each hMSC-based product should be evaluated. This cost could be assumed in the case of rare diseases with a low prevalence, but it seems quite difficult to extend the use of these products to a wide prevalent medical condition. Hence, the challenge in the manufacturing of these products does not only cover the standardization of quality, safety, and efficacy criteria, but also the optimization of their costs. 

There is not a universal protocol or universal definition on how technical requirements should be implemented throughout the manufacturing process, nor on the methods and analytical techniques, but each sponsor must design and validate a specific process for their product, guaranteeing the function and integrity of hMSCs and ensuring that the hMSC-based final product consistently meets its specifications. Then, all data about the defined manufacturing process must be submitted to regulatory authorities, which reviews the information and approves the protocol if appropriate. Therefore, sponsors must evaluate the cost-benefit of their process. Some alternatives to reduce costs could be the mass production of allogeneic doses, incorporation and adaptation of automatized systems, reduction of specific technical requirements implemented in intermediate hMSC-based products, and definition of minimum acceptance criteria for each batch of hMSC-based product. The costs could also be optimized by assessing other aspects outside the manufacturing process, such as the use or not of operating rooms for the product administration or considering the need of the patient hospitalization. 

Today, hMSCs are subject to numerous studies, both preclinical and clinical, to research and assess their efficacy and safety as treatment in different pathologies, and to determine aspects that have not yet been defined, such as their MoA, maximum dosage, and ideal routes of administration, among others. Furthermore, in order to define what exactly quality control and when it must be carried out is an important issue, since the number of these controls will affect the price of hMSC-based products, and thus their accessibility in the market. Therefore, the development of hMSC-based products is an area that is constantly evolving.

## Figures and Tables

**Table 1:** Specifications of hMSC-based final products before administration to a patient based on the primary criteria for quality.

Cell Characterization Criteria	Assays	Methods	Release Criteria	According to Different Frameworks	
				FDA	EMA
Identity	Morphology	Microscopy	Large cells similar in size to fibroblasts Cells that are spindle-shaped or slightly star-shaped, like fibroblasts. Large cell nuclei containing frequent large, dark granules (Occasionally binucleated) Uniform monolayer (absence of cell aggregates)	N/A	N/A
	Phenotypic markers	Flow cytometry, FACS analysis	(>90%) for CD105, CD73, CD90, (<5%) for CD45, CD34 and CD14	N/A	N/A
	Ability to differentiate	Stain	Differentiate into osteoblasts, adipocytes or chondroblasts	N/A	N/A
Purity	Endotoxin detection	LAL test	≤0.5 EU/ml	ICH Guideline Q4B Annex 14	
				USP: <85> Bacterial Endotoxins Test, USP 33 Reissue 21 CFR 600.3 21 CFR 610.9 Guidance for Industry Pyrogen and Endotoxins Testing: Questions and Answers (Jun 2012)	Eur. Ph. (2.6.14.) Monograph on Bacterial endotoxins EDQM 5.1.10. Guidelines for Using the Test for Bacterial Endotoxins
Potency	Paracrine secretion (cytokines)	ELISA HPLC	Specific by cytokine Concentration of kynurenine production at 24–48 h, previously separated for 24 h after its induction of the IDO system with interferon-gamma (INF-y) for the consumption of tryptophan	ICH Q6B	
				Guidance for Industry Potency Tests for Cellular and Gene Therapy Products (Jan 2011)	Guideline on Human Cell-Based Medicinal Products (May 2008) Guideline on potency testing of cell-based immunotherapy medicinal products for the treatment of cancer (2016)
Viability	Living/dead cell count	Trypan blue dye exclusion Acridine Orange / Propidium Iodide (AO/PI) Cell Viability Kit	≥80% (EMA) ≥70% (FDA)	USP <1046> Cell and Gene Therapy Products	Eur. Ph. (2.7.29.) Nucleated cell count and viability.
Proliferative capacity	Total cumulative population doubling	Total number of cellular divisions	No senescence	N/A	N/A
Genomic stability	Karyotype	FISH SKY SNP aCGH G-banding DAPI banding QFQ-banding (at least 20metaphases)	Absence of clonal chromosomal aberrations Presence of non-clonal chromosomal aberrations in ≤10% of metaphases analyzed	N/A	N/A
Microbiological quality control	Sterility test	Direct inoculation	Negative (no haze in the media)	ICH guideline Q4B Annex 8	
				21 CFR 610.12 – Sterility USP <71> Sterility Alternative methods possible under 21 CFR 610.9	Eur. Ph.: (2.6.27) Microbiological control of cellular products Eur. Ph.: (2.6.1.) Sterility Eur. Ph.: (5.1.6) Alternative methods for control of microbiological quality
	Mycoplasma test	Real-time PCR	Negative	USP <63> Mycoplasma Tests	Eur. Ph. (2.6.7.) Monograph Mycoplasmas EMA/410/01 rev.3
	Adventitious viruses (for allogeneic products)	In vitro adventitious viral agent test	Negative	ICH Topic Q 5 A (R1)	
				USP <1050.1> Offers Practical Approaches to ICH Q5A Viral Clearance Testing	Guideline on virus safety evaluation of biotechnological investigational medicinal products. 2006.

Abbreviations: FACS (Fluorescence-activated cell sorting); LAL (Limulus amebocyte lysate); ELISA (enzyme-linked immunosorbent assay); HPLC (high-pressure liquid chromatography); Eur. Ph. (European Pharmacopoeia); EU (Endotoxin Units); Food and Drug Administration (FDA); European Medicines Agency (EMA); USP (United States Pharmacopeia); European Directorate for the Quality of Medicines & HealthCare (EDQM); Fluorescence In Situ Hybridization (FISH); Spectral Karyotyping (SKY); Single Nucleotide Polymorphism Array (SNP); Array-Based Comparative Genomic Hybridization (aCGH); Giemsa banding (G- banding); 4’-6-diamidino-2-phenylindole (DAPI) banding.
